# *In Vitro* and *in Vivo* Antioxidant Properties of the Plant-Based Supplement Greens+™

**DOI:** 10.3390/ijms12084896

**Published:** 2011-08-03

**Authors:** Venket Rao, Bashyam Balachandran, Honglei Shen, Alan Logan, Leticia Rao

**Affiliations:** 1 Department of Nutritional Sciences, Faculty of Medicine, University of Toronto, 150 College Street, Toronto, Ontario, M5S 3E2, Canada; E-Mails: balchan20@yahoo.com (B.B.); Honglei.shen@utoronto.ca (H.S.); 2 Integrative Care Centre of Toronto, 3600 Ellesmere Road, Unit 4, Toronto, Ontario, M1C 4Y8, Canada; E-Mail: aclnd@cfs-fm.org; 3 Department of Medicine, University of Toronto & Calcium Research Laboratory, Division of Endocrinology & Metabolism, St. Michael’s Hospital, Toronto, Ontario, M5B 1W8, Canada; E-Mail: leticia.rao@utoronto.ca

**Keywords:** dietary supplements, polyphenols, antioxidants, liposomes, herbal supplement, greens+, oxidative stress

## Abstract

Dietary antioxidants play an important role against oxidation, an underlying mechanism in the incidence of chronic diseases. Greens+ is a commercially available preparation containing a variety of plant-derived ingredients. The aim of the current study was to evaluate the antioxidant potential of the methanolic extract of greens+ powder using *in vitro* and *in vivo* techniques. *In vitro* studies were conducted using a liposome model system to simulate biological cell membranes. Total antioxidant potential and polyphenol content of the herbal preparation was measured. For *in vivo* analysis, 10 healthy human subjects consumed either three or six teaspoons of greens+ per day for four weeks. Blood samples were analyzed at baseline and at the conclusion of the treatment period for total antioxidant capacity, polyphenol content, protein, lipid and LDL oxidation, and the level of glutathione peroxidase. Results showed that greens+ supplementation was well tolerated and increased serum antioxidant potential at higher levels of intake in a dose-dependent manner. HPLC analysis showed the presence of quercetin, apigenin, kaempferol and luteolin in the supplement. Plasma analysis indicated the presence of kaempferol only. A statistically significant (p < 0.05) reduction in protein and lipid oxidation was observed. Based on its antioxidant properties, the results suggest that greens+ might play a role in reducing the risk of chronic diseases involving a burden of oxidative damage.

## 1. Introduction

Oxidative damage to cellular biomolecules such as lipids, proteins and DNA is thought to play a crucial role in the incidence of several chronic diseases [1–5]. Flavonoids are a group of polyphenolic compounds found abundantly in the plant kingdom. Interest in the possible health benefits of flavonoids and other polyphenolic compounds has increased in recent years owing to their potent antioxidant and free-radical scavenging activities [6–12]. Greens+ is a commercially available herbal preparation containing various amounts of plant derived and other products that are potential sources of polyphenol antioxidants that are purported to be beneficial for human health. A list of ingredients in a single serving of greens+ in shown in Table 1. Although greens+ has been the subject of both clinical and experimental investigation [13–15], there have been no studies to evaluate its antioxidant properties. Therefore, the aim of the present study is to evaluate antioxidant potential of the herbal preparation greens+ using the *in vitro* and *in vivo* techniques. A clear understanding of the antioxidant properties of this herbal preparation will contribute towards evaluating its efficacy and make appropriate recommendation for its use.

## 2. Experimental Methods

### 2.1. Materials

*In vitro* experiments consisted of: (i) measuring the overall antioxidant potential in a methanolic extract of greens+ of various concentrations. The ABTS (2,2’-azino-bis(3-ethylbenzthiazoline-6-sulfonic acid) decoloration assay [16] was used. (ii) measuring the antioxidant potential of greens+ extract using a liposome model to simulate biological cell membrane. (iii) HPLC estimation of the polyphenols present in greens+. The greens+ herbal preparation used in the study was provided by Genuine Health, Toronto, Ontario. Polyphenols standards and trolox were obtained from Sigma Chemical Co., St. Louis, MO, USA.

### 2.2. *In Vitro* Study

#### 2.2.1. ABTS^+ •^ Decoloration Assay

The overall antioxidant potential of the greens+ expressed as trolox equivalent was estimated using the ABTS (2,2’-azino-bis(3-ethylbenzthiazoline-6-sulfonic acid) method [16]. Results are expressed in terms of nmol/L trolox equivalent.

#### 2.2.2. Liposomal Lipid Peroxidation

Multilamellar liposome membranes were prepared using 100 mg egg phosphatidyl choline in 20 mL of 2 mM phosphate buffer using the method as described by Balachandran and Rao [17]. They were used as a model system to simulate biological cell membranes to measure antioxidant properties of greens+. In 1 mL of Liposomal solution, lipid peroxidation was initiated by adding 100 μL of 1 mM FeSO_4_ in the presence of 100 μL of greens+ solution at various concentrations (0.5%, 1%, 1.5%, 2% and 2.5%). The final concentrations of liposome and FeSO_4_ in the incubation mixture were 5 mg/mL and 0.09 mM respectively. Lipid oxidation was measured by the thiobarbituric acid (TBA)-malondialdehyde (MDA) assay [18].

#### 2.2.3. Polyphenol Analysis in Green+ Herbal Preparation

One gram of greens+ powder was extracted with 20 mL 50% acetone and hydrolyzed with 5 mL of 6 M HCl for 1 h. The hydrolysate was cooled and centrifuged at 3,000 rpm for 15 min at room temperature. The supernatant was then neutralized with 10 N NaOH to pH 6.6 and diluted with distilled water. The diluted extract was passed through Sep-Pak C18 (Waters Corporation, Milford, MA, USA) cartridge column for solid phase extraction. The column was then eluted with 100% acetone.

#### 2.2.4. HPLC Analysis

The analysis was carried out according to the method of Hollman *et al.* [19]. Chromatographic separations were carried out on a μ-Bondapak C18 (Waters Corporation, Milford, MA, USA) column (3.9 × 150 mm, 10 μm) at 35 °C. A UV/Visible detector (Shimadzu, Kyoto, Japan) was used to detect polyphenols. The mobile phase consisted of solution A: 0.1 M NH_4_H_2_PO_4_, pH 4.4 and solution B: 0.1 M NH_4_H_2_PO_4_, pH 4.4 (20%), acetonitrile (60%), methanol (10%) and water (10%) with gradient 0–15% B, 0–5 min; 60% B, 5–30 min; 100% B, 30–50 min. The flow rate of the mobile phase was 1 mL/min. Ten microlitre was injected onto the column. The external standards of flavonoids such as quercetin, kaempferol, myricitin, luteolin and apigenin (Sigma Chemical Co., St. Louis, MO, USA) were used as reference standards.

### 2.3. *In Vivo* Study

#### 2.3.1. Evaluation of Plasma Polyphenols and Antioxidant Properties of Greens+ Herbal Preparation

##### 2.3.1.1. Subjects

The *in vivo* study involved (i) measuring serum total antioxidant potential; (ii) measuring serum lipid, protein and LDL oxidation; (iii) measuring erythrocyte glutathione peroxidase activity; (iv) HPLC measurement of the levels of plasma polyphenols. Ten healthy human subjects (Five men and five women), nonsmokers who were not taking any medication or vitamin supplements, were recruited into the study. Average age (years), weight (kg), and body mass index of the subjects were 29.3 ± 2.3, 72.0 ± 3.37, and 24.1 ± 0.70 respectively. The average systolic and diastolic pressure were in the normal range of 104 ± 1.63 and 66.5 ± 1.30 respectively. Subjects maintained their body weight during the study period.

##### 2.3.1.2. Study Design

**Figure 1.**
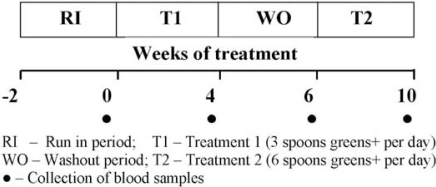
Study protocol used in the study.

Study protocol used in this study is shown in Figure 1. During the 2 weeks of the Run-In period (RI), subjects consumed their usual diets and, during washout as well as treatment periods, they refrained from smoking and taking vitamin supplements and maintained their usual diet and life style activities. Serum and plasma were separated from fresh blood, aliquoted, and stored at −70 °C for biochemical analysis. The Human Ethics Committee of the University of Toronto approved the study protocol.

#### 2.3.2. Polyphenol Analysis in Plasma after Greens+ Supplementation

For the analysis of polyphenols in plasma, the method as described previously for greens+ herbal preparation was used.

#### 2.3.3. Oxidative Biomarker Analyses

##### 2.3.3.1. Total Antioxidant Capacity *in Vivo*

Trolox equivalent antioxidant capacity in serum was measured by the spectrophotometric ABTS^+ •^ method [16] as in the *in vitro* procedures.

##### 2.3.3.2. Protein Oxidation

Protein oxidation was estimated by measuring the loss of reduced thiol (-SH) groups using 5,5’-dithio-bis(2-nitrobenzoic acid) (DTNB) assay [20]. Thiols were calculated using the extinction coefficient of 13.6 mM^−1^.

##### 2.3.3.3. Lipid Peroxidation

Malondialdehyde, as a measure of lipid peroxidation, was measured by the thiobarbituric acid-malondialdehyde (TBA-MDA) assay and reported as TBA reactive substances (TBARS) [18]. Results were calculated using ɛ_535_ 1.56 × 10^5^ M^−1^.

##### 2.3.3.4. LDL Oxidation

For LDL oxidation analysis, serum LDL was isolated by isoelectric precipitation with buffered heparin [21]. LDL cholesterol contents were estimated enzymatically [22] using Cholesterol Assay Kit (Sigma Chemical Company, St. Louis, MO, USA). MDA, as a measure of LDL oxidation, was estimated using the thiobarbituric acid (TBA) reaction. Results were expressed as TBA reactive substances (TBARS). Freshly prepared LDL samples were incubated with TBA at 95 °C, extracted with n-butanol. The absorbance of the butanol extract was read at 535 using spectrophotometer. Results were calculated using ɛ_535_ 1.56 × 10^5^ M^−1^.

##### 2.3.3.5. Determination of Glutathione Peroxidase in Erythrocytes

Glutathione peroxidase activity in erythrocytes was measured using the method of Pleban *et al.* [23]. The enzyme activity was measured as U/g of Hb.

### 2.4. Statistical Analysis

All calculations were performed using Excel 5.0 (Microsoft Corporation). The paired Student’s *t* test was used to compare the results of the RI and treatment groups. Values are reported as means ± SEM. A probability value less than 0.05 (p < 0.05) was considered statistically significant.

## 3. Results and Discussion

### 3.1. *In Vitro* Study

#### 3.1.1. Total Antioxidant Properties of Greens+^™^ Herbal Preparation

**Figure 2.**
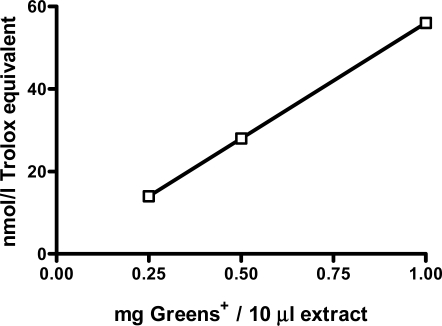
Dose dependent antioxidant effect of greens+ (nmol/L trolox equivalent). The results represent the mean of values obtained from three measurements.

Greens+ is a commercially available herbal supplement containing several botanical products that are considered as good sources of the polyphenols having antioxidant properties. The herbal preparation itself was never tested for its antioxidant properties. Results obtained in this study are shown in Figure 2. A dose-dependent linear antioxidant effect was observed with up to 1 mg of greens+ extract.

**Figure 3.**
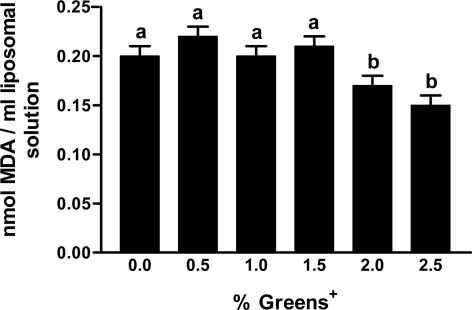
Effect of greens+ on FeSO_4_ induced lipid peroxidation using a liposome model. The results represent the mean ± SEM of values obtained from three measurements. Different corresponding letters indicate significant differences at p < 0.05 by Duncan’s test.

Based on this observation, the antioxidant potential of greens+ was evaluated in a liposomal lipid peroxidation model. Liposomes are commonly used to simulate cell membranes. As shown in Figure 3, a significant (p < 0.05) reduction in the production of malondialdehyde (MDA), which is a measure of lipid peroxidation, was observed at higher concentrations (2% and 2.5%) of greens+ compared to lower concentrations (0.5%, 1% and 1.5%). The reduction of lipid peroxidation in the liposomes, in the presence of higher concentrations of greens+, suggest the ability of the polyphenols present in the herbal preparation to protect membrane lipids form oxidation.

#### 3.1.2. Identification and Quantification of Polyphenols in Greens+

HPLC procedure was used to detect the presence of polyphenols present in greens+ and to quantify them. Polyphenol composition of the herbal preparation is shown in Table 2. Quercetin, apigenin, kaempferol and luteolin were the four major polyphenols detected. This study was the first to show the presence of these polyphenols in greens+.

### 3.2. *In Vivo* Study

For the *in vivo* estimation of the antioxidant potential of greens+, ten healthy human subjects consumed either three or six teaspoons of the herbal preparation per day for four weeks. Blood samples were collected, processed and analyzed for the presence of polyphenols, total antioxidant potential, lipid, protein and LDL oxidation, and glutathione peroxidase activity.

#### 3.2.1. Effect of Ingesting Greens+ on Body Weight, BMI and Blood Pressure

No changes were observed in the body weights, BMI and blood pressure of the subjects after oral supplementation of greens+ at two different doses for four weeks. These observations suggest no adverse effects of ingesting greens+ even up to six teaspoons per day for four weeks.

#### 3.2.2. Effect of Ingesting Greens+ on Total Antioxidant Capacity

**Figure 4.**
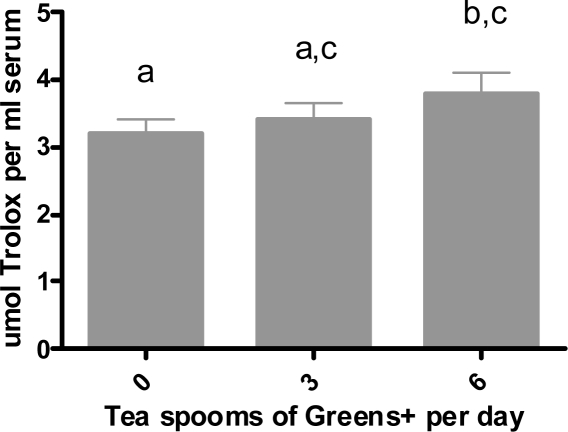
Dose dependent effect of greens+ on serum total antioxidant capacity (nmol Trolox equivalent/mL serum). The results represent the mean ± SEM of values obtained from three measurements. Different letters indicate significant differences at p < 0.05 by Duncan’s test.

Results are shown in Figure 4. Although, the consumption of three teaspoons of greens+ per day for four weeks increased the total antioxidant capacity, it was not statistically different from the baseline. However, maximum antioxidant capacity was observed after six teaspoons of ingestion per day for four weeks. This increase from the baseline was statistically significant (p < 0.05).

#### 3.2.3. Effect of Ingesting Greens+ on Lipid Oxidation

**Figure 5.**
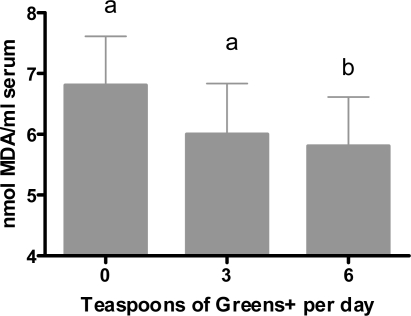
Dose dependent effect of greens+ on serum lipid oxidation (nmol MDA/mL serum). The results represent the mean ± SEM of values obtained from three measurements. Different corresponding letters indicate significant differences at p < 0.05 by Duncan’s test.

Figure 5 shows the results of ingesting greens+ on lipid oxidation. As can be seen, a statistically significant reduction in serum MDA was observed after four weeks of treatment at the consumption level of six teaspoons. Although consumption of three teaspoons of greens+ for four weeks showed a minor increase in serum lipid MDA, this change was not statistically different from the baseline. This may have been due to the small number of subjects and a relatively shorter period of intake.

#### 3.2.4. Effect of Ingesting Greens+ on Protein Oxidation

**Figure 6.**
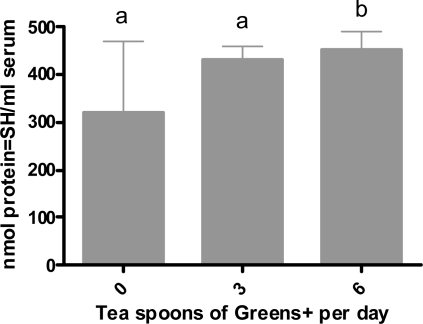
Dose dependent effect of greens+ on serum protein oxidation (nmol protein-SH/mL serum). The results represent the mean ± SEM of values obtained from three measurements. Different corresponding letters indicate significant differences at p < 0.05 by Duncan’s test.

Results are shown in Figure 6. Similar to the effects on lipid oxidation, consuming Greens+ at the level of six teaspoons per day for four weeks significantly increased the levels of protein thiols indicating a significant reduction in protein oxidation. Although the levels of protein thiols for three-teaspoon consumption showed an increase over the baseline, it was not statistically significant.

#### 3.2.5. Effect of Ingesting Greens+ on Erythrocyte Glutathione Peroxidase

**Figure 7.**
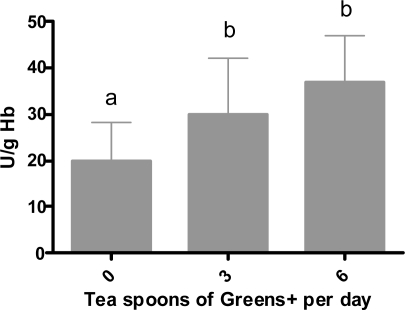
Dose-dependent effect of greens+ on erythrocyte glutathion peroxidase activity (U/g Hb). The results represent the mean ± SEM of values obtained from three measurements. Different corresponding letters indicate significant differences at p < 0.05 by Duncan’s test.

Results are shown in Figure 7. A statistically significant increase in the activity of erythrocyte glutathione peroxidase was observed after consuming the herbal preparation for four weeks at both three and six teaspoons per day.

A study was undertaken to investigate the *in vitro* and *in vivo* antioxidant properties of the herbal preparation greens+. The liposome model was used in the *in vitro* studies to simulate a biological cell membrane. Greens+ showed a dose-dependent trolox equivalent antioxidant capacity under the *in vitro* conditions. A similar effect on the total antioxidant potential was also seen in the serum of human subjects consuming greens+ for a period of four weeks. HPLC analysis of greens+ showed the presence of four major polyphenols: quercetin, apigenin, kaempferol and luteolin. Since the powdered greens+ preparation contains several plant-derived products that are evidently significant sources of antioxidant polyphenols, the increase in the serum antioxidant capacity, following greens+ consumption, could be related to the absorption of the polyphenols. Kaempferol was found to be the only major polyphenol present in the human plasma at higher concentration (six teaspoons) after four weeks of consumption. Some of the other polyphenols found in greens+ were undetected in the plasma of subjects. This may be due to the accumulation of flavonoids mainly as glucoronide and sulfate conjugates in blood plasma [24]. Nevertheless, the conjugated metabolites of flavonoids may play a role in the antioxidant defense of blood plasma. Terao [24] found that oral administration of epicatechin and quercetin resulted in the accumulation of the glucoronide and sulphate conjugates in rat blood plasma.

The liposome model system used in this study also showed a significant antioxidant capacity at higher concentrations of ingestion. Cao *et al.* [25] demonstrated that the consumption of strawberries, spinach, red wine or vitamin C increased the antioxidant capacity of serum in elderly women. Other studies have shown that many flavonoids present in plants have the ability to scavenge peroxyl, alkyl-peroxy radicals, superoxide hydroxyl radicals and peroxynitrile in aqueous and organic environment.

The consumption of greens+ by healthy human subjects showed a significant increase in the levels of reduced thiols indicating a decrease in the level of oxidized proteins in the serum. The damage caused to enzymes via protein oxidation is significant in discussions regarding human health. The activity of metabolic enzymes that otherwise play a crucial role in the detoxification of carcinogens and repair the damaged DNA may be inhibited, the net result being a contribution to metabolic disorders and increased risk of chronic disease [26–29]. Also, oxidation of proteins present in cell membrane will alter both the structure and functional characteristics of the cells.

Serum MDA, indication of lipid peroxidation, was reduced significantly with the consumption of greens+. This effect is likely related to the presence of polyphenols. Plant phenolic compounds trap chain-initiating radicals at the interface of the membrane, thus preventing the progression of the radical chain reaction. Additionally, the phytochemicals such as the polyphenols can chelate the transition metal ions responsible for the generation of reactive oxygen species and therefore inhibit the initiation of the lipoxygenase reaction [30].

Greens+ administration did not change LDL oxidation as measured by LDL malondialdehyde. This could be ascribed to the presence of more water-soluble polyphenols that might have been absorbed by the protein-rich HDL fraction. Polyphenols have been shown to have high affinity for proteins [31]. Other studies have also shown similar lack of protection against LDL oxidation by the consumption of grape extract [32]and tea [33–35].

Results obtained in the present study also showed an increase in the erythrocyte glutathione peroxidase activity during greens+ supplementation.

## 4. Conclusions

In summary, the greens+ formulation includes a variety of plant-derived ingredients with polyphenolic antioxidants that have been identified via *in vitro* testing. The results of the *in vivo* arm of the study suggest that one or more of these phytochemicals are well absorbed, retain their antioxidant properties *in vivo* and lower oxidative stress at an intake level of six teaspoons per day for four weeks. The trend observed with three teaspoons could be due to shorter period of intake and smaller number of subjects. A long-term study with larger number of subjects should be undertaken to confirm the effect of low level intake of greens+ on oxidative stress. Overall, the results suggest a potentially important role of greens+ in reducing oxidative stress and as such, it may play a long-term role in the prevention of chronic diseases.

## Figures and Tables

**Table 1.** Ingredients in a single serving of greens+.

**Ingredients per 8.5 g serving of supplement**		
Phosphatide complex (26% phosphatidyl choline from 97% oil-free lecithin)	2,171	mg
Organic barley, alfalfa and wheat grass, and red beet powders	1,543	mg
Spirulina	1,450	mg
Apple fiber powder	1,033	mg
Japanese chlorella (cracked cell)	383	mg
Organic soy sprout powder	383	mg
Organic whole brown rice powder	383	mg
Stevia leaf powder	225	mg
Eight non-dairy bacterial cultures containing Lactobacilli and bifidobacteria (2.5 billion per serving) in a special base of fructo-oligosaccharides (FOS)	200	mg
Royal jelly (5% 10-HDA)	150	mg
Bee pollen powder	150	mg
Licorice root extract standardized to 10% glycyrrhizin (5:1 = 580 mg)	116	mg
Acerola berry extract standardized to 18% Vitamin C	115	mg
Siberian ginseng extract standardized to 0.8% eleutherosides (28:1 = 1,680 mg)	60	mg
Milk thistle extract standardized to 80% silymarin (15:1 = 900 mg)	60	mg
Organic Atlantic dulse powder	33	mg
Ginkgo biloba extract standardized to 24% ginkgo flavonglycosides and 6% terpene lactones (50:1 = 1,000 mg)	20	mg
Japanese green tea extract standardized to 90% polyphenols (20:1 = 300 mg)	15	mg
European bilberry extract standardized to 25% anthocyanidins (100:1 = 1,000 mg)	10	mg
Full spectrum grape extract standardized to 95% proanthocyanidins and 500 ppm Resveratrol (500:1 = 2,500 mg)	5	mg

**Table 2.** Polyphenol composition of greens+ herbal preparation.

**Flavonoid**	**Amount (μg/g)**
Quercetin	177.25
Apigenin	133.31
Kaempferol	98.56
Luteolin	58.28

## References

[1] Simonian NA, Coyle JT (1996). Oxidative stress in neurodegenerative diseases. Annu. Rev. Pharmacol. Toxicol.

[2] Dhalla NS, Temsah RM, Netticadan T (2000). Role of oxidative stress in cardiovascular diseases. J. Hypertens.

[3] Van’t Veer P, Jansen MC, Klerk M, Kok FJ (2000). Fruits and vegetables in the prevention of cancer and cardiovascular disease. Public Health Nutr.

[4] Bokov A, Chaudhuri A, Richardson A (2004). The role of oxidative damage and stress in aging. Mech. Ageing Dev.

[5] Madamanchi NR, Vendrov A, Runge MS (2005). Oxidative stress and vascular disease. Arterioscler. Thromb. Vasc. Biol.

[6] Bravo L (1998). Polyphenol: Chemistry, dietary sources, metabolism, and nutritional significance. Nutr. Rev.

[7] Heim KE, Tagliaferro AR, Bobilya DJ (2002). Flavonoid antioxidants: Chemistry, metabolism and structure-activity relationships. J. Nutr. Biochem.

[8] Pier-Giorgio P (2000). Flavonoids as antioxidants. J. Nat. Prod.

[9] Rice-Evans C, Miller N, Paganga G (1997). Antioxidant properties of phenoilc compounds. Trends Plant Sci.

[10] Sealbert A, Johnson J, Saltmarsh M (2005). Polyphenols: Antioxidants and beyond. Am. J. Clin. Nutr.

[11] Ross JA, Kasum CM (2002). Dietary flavonoids: Bioavailability, metabolic effects, and safety. Ann. Rev. Nutr.

[12] Rice-Evans CA, Miller NJ, Paganga G (1996). Structure-antioxidant activity relationships of flavonoids and phenolic acids. Free Radic. Biol. Med.

[13] Berardi J, Logan A, Rao AV (2008). Plant based dietary supplement increases urinary pH. J. Int. Soc. Sports Nutr.

[14] Bloom H (2004). Effect of greens+: A randomized, controlled trial. Can. J. Diet Pract. Res.

[15] Rao LG, Balachandran B, Rao AV (2008). Polyphenol extract of greens+TM nutritional supplement stimulates bone formation in cultures of human osteoblast-like SaOS-2 cells. J. Herb. Pharmacother.

[16] Re R, Pellegrini N, Proteggente A, Pannala A, Yang M, Rice-Evans C (1999). Antioxidant activity: Applying an improved ABTS radical cation decolorization assay. Free Radic. Biol. Med.

[17] Balachandran B, Rao AV (2003). Time dependent uptake and antiperoxidative potential of lycopene in multilamellar liposomes. Food Res. Int.

[18] Draper HH, Squires EJ, Mahmood H, Wu J, Agarwal S, Hadley MA (1993). Comparative evaluation of thiobarbituric acid methods for the determination of malondialdehyde in biological materials. Free Radic. Biol. Med.

[19] Hollman PCH, van Trijp JMP, Buysman MNCP (1996). Fluorescence detection of flavonols in HPLC by postcolumn chelation with aluminum. Anal. Chem.

[20] Hu ML (1994). Measurement of protein thiol groups and glutathione in plasma. Meth. Enzymol.

[21] Wieland H, Seidel D (1983). A Simple specific method for precipitation of low density lipoproteins. J. Lipid Res.

[22] Allain CA, Poon LS, Chan CSG, Richmond W, Fu PC (1974). Enzymatic determination of total serum cholesterol. Clin. Chem.

[23] Pleban PA, Munyani A, Beachum J (1982). Determination of selenium concentration and glutathione peroxidase activity in plasma and erythrocytes. Clin. Chem.

[24] Terao J (1999). Dietary flavonoids as antioxidants *in vivo*: Conjugated metabolites of (−)-epicatechin and quercetin participate in antioxidative defense in blood plasma. J. Med. Invest.

[25] Cao G, Russell RM, Lischner N, Prior RL (1998). Serum antioxidant capacity is increased by consumption of strawberries, spinach, red wine or vitamin C in elderly women. J. Nutr.

[26] Frei B (2004). Efficacy of dietary antioxidants to prevent oxidative damage and inhibit chronic disease. J. Nutr.

[27] Descamps-Latscha B, Witko-Sarsat V, London GM, Nguyen-Khoa T, Nguyen AT, Gausson V, Mothu N, Jungers P (2005). Oxidation protein products as risk factors for atherosclerotic cardiovascular events in nondiabetic predialysis patients. Am. J. Kidney Dis.

[28] Hall NC, Gillan AH (1979). Effects of antirheumatic drugs on protein sulphydral reactivity of human serum. J. Pharm. Pharmacol.

[29] Kadota K, Yui Y, Hattori R, Murohara Y, Kawai C (1991). Decreased sulfhydryl groups of serum albumin in coronary artery disease. Jpn. Circ. J.

[30] Hider RC, Liu ZD, Khodr HH (2001). Metal chelation of polyphenols. Meth. Enzymol.

[31] Diniz A, Escuder-Gilabert L, Lopes NP, Villanueva-Camanas RM, Sagrado S, Medina-Hernandez MJ (2008). Characterization of interactions between polyphenolic compounds and human serum proteins by capillary electrophoresis. Anal. Bioanal. Chem.

[32] Rao AV, Shen H, Agarwal A, Yatcilla MT, Agarwal S (2000). Bioabsorption and *in vivo* antioxidant properties of grape extract biovin®: A human intervention study. J. Med. Food.

[33] Van het Hof KH, de Boer HSM, Wiseman SA, Lien N, Wetstrate JA, Tijburg LBM (1997). Consumption of green or black tea does not increase resistance of low-density lipoprotein to oxidation in humans. Am. J. Clin. Nutr.

[34] McAnlis GT, McEneny J, Pearce J, Young IS (1998). Black tea consumption does not protect low density lipoprotein from oxidative modification. Eur. J. Clin. Nutr.

[35] Princen HMG, van Duyvenvoorde W, Buytenhek R, Blonk C, Tijburg LBM, Langius JAE, Meinders AE, Pijl H (1998). No effect of consumption of green and black tea on plasma lipid and antioxidant levels and on LDL oxidation in smokers. Arterioscler. Thromb. Vasc. Biol.

